# Draft Genome Sequence of *Indibacter alkaliphilus* Strain LW1^T^, Isolated from Lonar Lake, a Haloalkaline Lake in the Buldana District of Maharashtra, India

**DOI:** 10.1128/genomeA.00513-13

**Published:** 2013-07-18

**Authors:** Ajit Singh, Pramod Kumar Jangir, Rakesh Sharma, Aditya Singh, Anil Kumar Pinnaka, S. Shivaji

**Affiliations:** CSIR–Institute of Genomics and Integrative Biology, Chandigarh, Indiaa; CSIR–Institute of Microbial Technology, Chandigarh, Indiab; CSIR–Centre for Cellular and Molecular Biology, Habsiguda, Hyderabad, Andhra Pradesh, Indiac

## Abstract

We report the 5.0-Mb genome sequence of *Indibacter alkaliphilus* strain LW1^T^, isolated from a haloalkaline crater lake in the Buldana district, Maharashtra, India.

## GENOME ANNOUNCEMENT

*Indibacter alkaliphilus* strain LW1^T^ was isolated from a water sample from Lonar Lake, a haloalkaline crater lake situated in the Buldana district of Maharashtra, India. It is a nonmotile, rod-shaped, Gram-negative bacterium (1). A 16S rRNA gene phylogenetic analysis revealed members of the genera *Belliella* and *Aquiflexum* to be the nearest neighbors of *Indibacter alkaliphilus* LW1^T^, with similarity values between 91.8 and 92.3%.

The genome of *Indibacter alkaliphilus* LW1^T^ was sequenced using two platforms, an Illumina HiSeq 1000 instrument (Illumina, Inc.) and an IonTorrent personal genome machine (PGM) (Life Technologies Corporation, CA). Illumina HiSeq sequencing yielded 2,525,254,924 bases in 25,002,524 reads, which is 503× coverage of the genome. Assembly of the raw reads was done using the CLC Genomics Workbench (v. 6.0.2; CLC bio, Denmark), which yielded a genome sequence of 5,020,409 bp in 64 contigs. Sequencing using the IonTorrent PGM yielded 114,661,689 bp in 266,959 reads, which is 209× coverage of the genome. The assembly of the sequencing data was done using a GS De Novo assembler (v. 2.5). Assembly yielded a genome sequence of 5,032,414 bp in 86 contigs. Assembled data from both the platforms were aligned and merged using the AMOS package (a modular, open-source whole-genome assembler, v. 3.1; http://sourceforge.net/projects/amos). This final assembly yielded a genome sequence of 5,036,047 bp in 56 contigs, with the largest contig being 587,558 bp long and the smallest contig being 587 bp long. The calculated G+C content of the genome was 39.68 mol%. RAST (rapid annotation using subsystem technology) (2) was used for the annotation of the genome of *Indibacter alkaliphilus* LW1^T^, tRNA was predicted by tRNAscan-SE (v. 1.23) (3), and rRNA genes were predicted by RNAmmer (v. 1.2) (4).

The annotated genome consists of 4,656 protein-coding genes and 44 RNA genes. Among the genes, 131 genes were found to be involved in membrane transport, including genes for 5 tripartite ATP-independent (TRAP) transporters, 7 cation transporters, and 11 uni-, sym-, and antiporters. Also, there were 48 genes involved in resistance against antibiotics and toxic compounds, 12 genes for intracellular resistance, and 81 genes for different stress responses, including 12 heat-shock, 3 cold-shock, and 12 periplasmic stress-response genes.

### Nucleotide sequence accession numbers.

The draft genome sequence of *Indibacter alkaliphilus* strain LW1^T^ has been deposited in DDBJ/EMBL/GenBank under the accession number ALWO00000000. The version described in this paper is the second version, ALWO02000000.
